# Rupture simultanée du ligament croisé antérieur et du ligament patellaire: à propos d'un cas

**DOI:** 10.11604/pamj.2016.23.20.8621

**Published:** 2016-01-28

**Authors:** Abdessalam Achkoun, Khalid Houjairi, Omar Quahtan, Jalal Hassoun, Mohamed Arssi, Mohamed Rahmi, Abdelhak Garch

**Affiliations:** 1 Service de Traumatologie-Orthopédie, Pavillon 32, Centre Hospitalier Universitaire Ibn Rochd, 1, rue des Hôpitaux, Quartier des hôpitaux 20360, Casablanca, Maroc

**Keywords:** Tendon patellaire, ligament croisé antérieur, rupture, reparation, Patellar tendon, anterior cruciate ligament, rupture, repair

## Abstract

La rupture simultanée du tendon rotulien et du ligament croisé antérieur est une lésion relativement rare. Son diagnostic peut facilement manquer lors de l'examen initial. Les options de traitement incluent la réparation immédiate du tendon rotulien avec soit la reconstruction simultanée ou différée de ligament croisé antérieur. Nous rapportons le cas d'une rupture combinée du tendon rotulien et du ligament croisé antérieur chez un jeune footballeur de 22 ans. Une approche de traitement en deux temps a été effectuée avec un excellent résultat fonctionnel.

## Abstract (en)

Simultaneous rupture of both the patellar tendon and the anterior cruciate ligament is a relatively rare injury. Its diagnosis can easily be missed during the initial examination. Treatment options include immediate repair of the patellar tendon with either simultaneous or delayed reconstruction of the ACL. We present the case of a combined rupture of the patellar tendon, the anterior cruciate ligament in a 22-year old footballer. A two-stage treatment approach was performed with an excellent functional outcome.

## Introduction

La rupture isolée du LCA est une lésion fréquente, tandis que la rupture isolée du ligament patellaire est moins observée. La rupture traumatique simultanée du ligament croisé antérieur et du ligament patellaire est rarement rapportée dans la littérature de diagnostic souvent méconnue et difficile, survient le plus souvent dans le cadre d'un traumatisme à haute énergie (poly traumatisme, accident de sport grave…). A travers un cas de rupture simultanée du ligament croisé antérieur et du ligament patellaire chez un patient de 22 ans, nous allons souligner la difficulté du diagnostic et de la prise en charge thérapeutique ainsi que l'intérêt d'une rééducation précoce.

## Patient et observation

**Figure 1:**
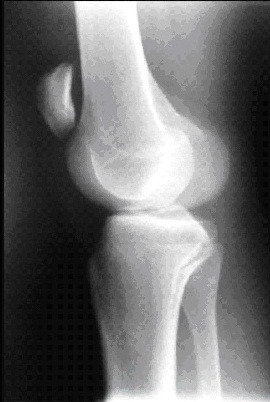
Aspect radiologique de la Patella Alta

**Figure 2:**
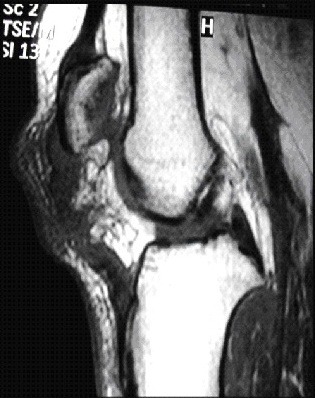
Coupe sagittale confirmant la rupture du LCA+LP

**Figure 3:**
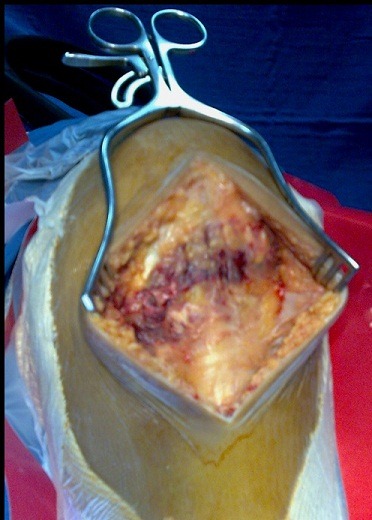
Aspect per opératoire objectivant la rupture du ligament patellaire

**Figure 4:**
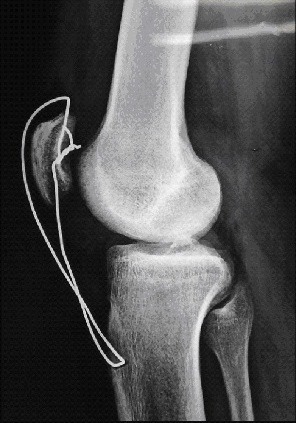
Aspect radiologique postopératoire montrant le cerclage métallique

Nous rapportons le cas d'un jeune footballeur de 22 ans, sans antécédents pathologiques notables (absence de maladies pré disposantes: LED, diabète, goutte, hyperparathyroïdisme notamment dans le cadre d'une IRC, rhumatisme inflammatoire ou obésité); qui a présenté suite a un accident de sport (torsion du membre inférieur gauche en appui, bloqué au sol) une instabilité immédiate du genou gauche avec sensation de craquement. Le patient a été contraint d'arrêter toute activité sportive. L'examen physique a montré une augmentation du volume du genou gauche avec déficit absolu d'extension active et impossibilité d'appui sur le membre inférieur, un choc rotulien positif, une ascension de la Patella et un hiatus sous patellaire. A côté de l'atteinte évidente du mécanisme extenseur le patient présentait également un tiroir antérieur à 90° de flexion du genou ainsi qu'un signe de Lachmann-Trillat positif; une ponction articulaire a pu retirer 70cc de liquide hématique. La radiographie standard a noté une Patella Alta gauche (Figure 1), l'IRM a confirmé le diagnostic en mettant en évidence une rupture complète du ligament patellaire et du LCA (Figure 2). La prise en charge chirurgicale a été entreprise en deux temps,dans un premier temps, la réparation du ligament patellaire (Figure 3) par suture termino-terminale associée à un cadrage métallique (Figure 4). En postopératoire immédiat, le patient a été immobilisé dans une attelle amovible en extension pendant 6 semaines. Une rééducation douce et progressive a été entamée immédiatement. Dans un second temps, la reconstruction du LCA avec les tendons ischio-jambiers et fixation par vis d'interférence (technique DIDT (droit interne et demi-tendineux)). L'appui a été immédiat en post opératoire à l'aide de 2 cannes, le travail en extension active a été débuté dès le lendemain et durant les 15 premiers jours suivants. 6 mois après l'intervention chirurgicale l'examen clinique du genou gauche a retrouvé une flexion à 140° et une extension à 0°, le test de Lachman s'est révélé négatif et le testing musculaire a montre que le quadriceps gauche est légèrement atrophie de 1 cm par rapport au droit. Le patient a repris ses activités sportives 2 mois après, soit 8 mois du postopératoire.

## Discussion

La rupture simultanée du LCA et du ligament patellaire est peu fréquente, survenant essentiellement chez des patients de sexe masculin entre 15 et 30 ans, le plus souvent suite à d'un traumatisme à haute énergie (poly traumatisme, accident de sport grave…) [1]. Elle est favorisée par les microtraumatismes répétés, les tendinopathies (genou du sauteur, maladie de Sin ding Larsen Johannsen), les causes iatrogènes (infiltrations de corticoïdes, chirurgies du genou.) [2]. Dans la littérature nous n'avons pu recenser que 9 cas similaires, trois d'entres eux seulement associaient une rupture simultanée du LCA et du ligament patellaire, tandis que les autres présentaient également une rupture du ligament LLI associée a celle du LCA et du ligament patellaire [3–5]. L'association a été au départ méconnu chez cinq des neufs cas auparavant mentionnés. Dans 3 cas, la rupture du ligament patellaire a été découverte fortuitement en per opératoire lors de l'exploration chirurgicale pour la réparation de la rupture du LCA ou du LLI [5, 6]. Quant a la rupture du LCA, elle a été méconnue dans 2 cas et découverte lors de la réparation du ligament patellaire [4]. La survenue exceptionnelle d'une rupture simultanée du LCA et du ligament patellaire, l'hémarthrose précoce, la douleur associée, les similarités dans le mécanisme de blessures et la non utilisation de l'IRM en post traumatique immédiat ont contribué à la méconnaissance du diagnostic dans ces cas. Dans notre situation le diagnostic a pu être posé précocement, cliniquement, devant le déficit d'extension active du genou, la dépression sous patellaire et le signe de Lachman positif, confirmé par la radiographie standard (Patella Alta) et l'IRM. Le choix du moment de la réparation du ligament patellaire et la reconstruction du LCA est crucial dans le cas des lésions associées [7]. Les réparations combinées du ligament patellaire et du LCA se sont souvent compliquées d'arthrofibrose donc il est communément recommandé que la suture du ligament patellaire soit immédiate car La réparation des formes négligées (après 6 semaines) est difficile, du fait de la rétraction du quadriceps et de l'ascension de la rotule et expose souvent a une amyotrophie du quadriceps ainsi qu'une limitation de la flexion du genou [7, 8]. La majorité des auteurs utilise des sutures termino-terminales renforcées par différentes plasties avec ou sans cadrage par fil métallique ou par matériel prothétique comme le PDS L'immobilisation postopératoire est préconisée par la plupart des auteurs, celle-ci peut se faire par une genouillère, une attelle plâtrée ou une orthèse amovible pendant 3 à 6 semaines. La rééducation doit être précoce par mobilisation passive du genou dès le 1^er^ jour postopératoire en tenant compte de la stabilité et de la solidité de la réparation chirurgicale. Ce travail passif sera relayé par une rééducation active [9]. Par contre la reconstruction du LCA est retardée afin d’éviter la phase inflammatoire. Il faut intervenir sur un genou froid, sec, indolore; la disparition de lœdème, l'amélioration des amplitudes des mouvements du genou et la restitution de l'extension du genou sont des éléments qui oriente limitent le risque d'arthrofibrose et une patella Baja (rotule trop basse) en post opératoire [10]. La reconstruction du LCA par autogreffe du tendon rotulien n'est pas envisageable dans ces cas, c'est l'autogreffe des tendons de la patte d'oie qui est recommandée tenant compte de la facilité d'exécution et de la récupération fonctionnelle rapide du genou sans pour autant fragiliser l'appareil extenseur [9, 10]. Cette intervention nécessite une rééducation très prudente pendant les 2 mois qui suivent l'opération. La marche avec appui est autorisée dès le lendemain de l'opération en gardant le genou en extension et en s'aidant de deux cannes. Des mouvements passifs de flexion, sans appui, sont possibles, en s'arrêtant lorsque les douleurs apparaissent. Trois semaines après l'intervention, les cannes sont supprimées et la rééducation peut être commencée. Elle a, pour premier but, l'obtention d'une bonne mobilité: l'extension complète doit être obtenue et la flexion doit être égale ou supérieure à 90° trois semaines plus tard, soit six semaines après l'opération [11, 12].

## Conclusion

Pour conclure, cette association doit être suspectée devant tout traumatisme aigu du genou présentant une laxité ligamentaire sagittale associée à une hémarthrose importante avec ou sans atteinte apparente de l'appareil extenseur. Une évaluation clinique minutieuse ainsi qu'une IRM peuvent fournir à l'opérateur les informations nécessaires pour planifier et choisir le traitement le plus approprié.
